# Intracranial lesion increase under anti-tuberculous therapy

**DOI:** 10.1590/0037-8682-0112-2020

**Published:** 2020-06-01

**Authors:** Handan Alay, Ayşe Albayrak

**Affiliations:** 1 Ataturk University, Faculty of Medicine, Department of Infectious Diseases and Clinical Microbiology, Erzurum, Turkey.

**FIGURE 1:**
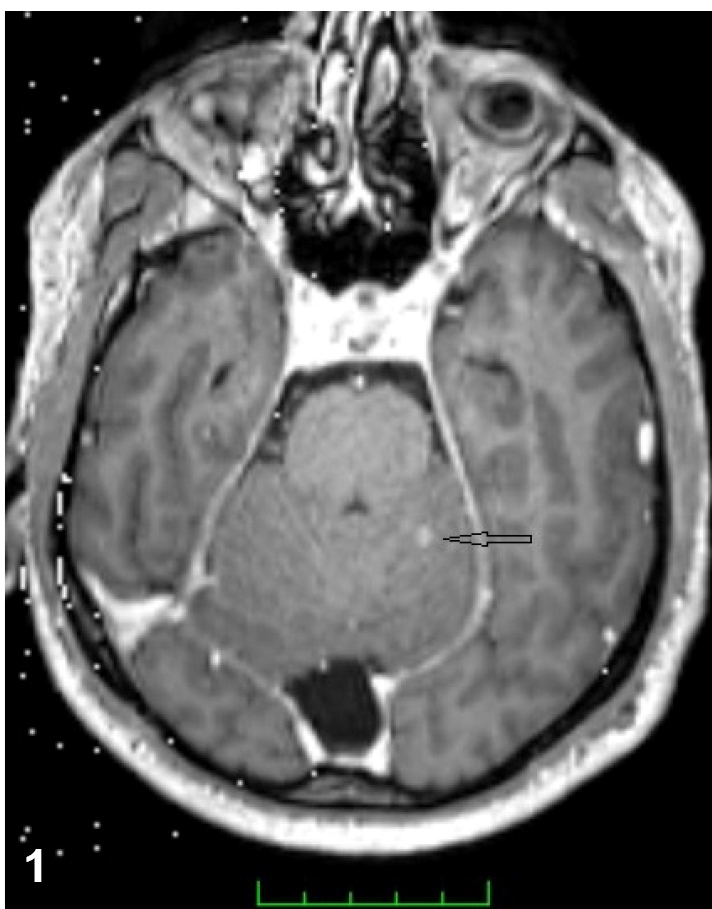
A millimetric contrast-enhancing tuberculoma focus in the left cerebellar hemisphere.

**FIGURE 2:**
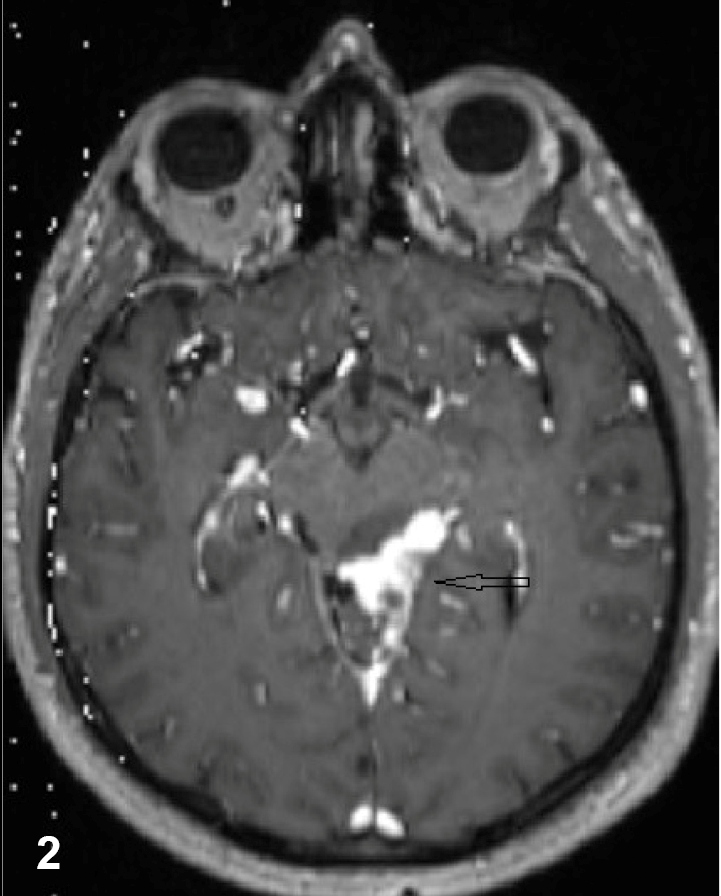
Increased linear enhancement suggesting basilar meningitis in the basilar cisterns.

**FIGURE 3:**
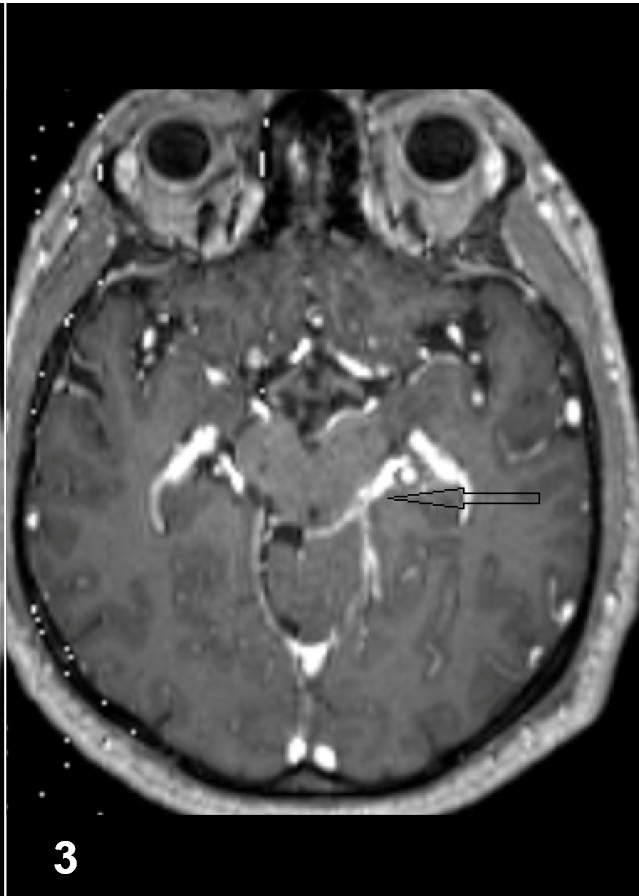
Decreased contrast enhancement in the basal cisterns.

A 43-year-old man presented to our clinic due to headache persisting for the previous 15 days, and with fever, vomiting, and altered consciousness for two days. At physical examination, the patient was somnolent, and signs of meningeal irritation were positive. Cerebrospinal fluid (CSF) analysis revealed microprotein: 138 mg/dL, chloride: 120 mmol/L, and glucose: 32 mg/dL (simultaneous blood sugar: 128 mg/dL). The headache persisted on the seventh day of anti-microbial therapy. Magnetic resonance imaging (MRI) revealed a millimeter-scale cerebellar lesion (Figure 1). PCR for *Mycobacterium tuberculosis* and Quantiferon tests of CSF were positive. The patient was commenced on isoniazid, 300 mg/day, rifampicin 600 mg/day, ethambutol 2 g/day and pyrazinamide 2 g/day. CSF culture testing over the first month of treatment was reported as *M. tuberculosis*, susceptible to anti-tuberculous drugs. In the second month of treatment, the patient was reexamined, complaining of severe headache. MRI of the brain revealed an increased number of lesions and basilar occlusion (Figure 2). Moxifloxacin and anti-edematous therapy were added to four-drug anti-tuberculous therapy. The symptoms resolved in the third month of five-drug anti-tuberculous therapy, and treatment was maintained with two-drug anti-tuberculous therapy. Improvement was recorded by MRI in the fifth month of treatment (Figure 3).

Central nervous system tuberculosis is a rare form of tuberculosis in immunocompetent individuals^1^. It can lead to excessive tuberculosis protein release in association with basilar obliteration, particularly in tuberculous meningitis, in patients receiving anti-tuberculous therapy. This protein can lead to tuberculoma expansion, or to identification of previously unseen tuberculomas^2^ ^,^ ^3^.
